# Primary Diffuse Leptomeningeal Melanomatosis Leading to Raised Intracranial Pressure in a Pediatric Patient

**DOI:** 10.1002/ccr3.9705

**Published:** 2025-01-26

**Authors:** Shiva Sareh, Zohreh Habibi, Mohammad Vasei, Moeinadin Safavi, Alieh Safari Sharari, Neda Pak, Golazin Shahbodagh khan, Mahmoudreza Ashrafi, Morteza Heidari

**Affiliations:** ^1^ Resident of Pediatrics, Children's Medical Center, Tehran University of Medical Sciences Tehran Iran; ^2^ Department of Neurosurgery School of Medicine, Children's Medical Center,Tehran University of Medical Sciences Tehran Iran; ^3^ Gene Therapy Research Center Digestive Disease Research Institute, Shariati Hospital, Tehran University of Medical Sciences Tehran Iran; ^4^ Department of Pathology and Laboratory Medicine Children's Medical Center Tehran Iran; ^5^ Department of Pediatrics, Pediatric Hematology and Oncology School of Medicine Children's Medical Center Tehran Iran; ^6^ Department of Radiology School of Medicine Children's Medical Center Tehran Iran; ^7^ Department of Pediatrics, Pediatric Neurology Fellowship School of Medicine Children's Medical Center Tehran Iran; ^8^ Department of Pediatrics, Professor of Pediatric Neurology School of Medicine Children's Medical Center Tehran Iran; ^9^ Department of Pediatrics, Division of Pediatric Neurology Children's Medical Center, Pediatrics Center of Excellent, Tehran University of Medical Sciences Tehran Iran

**Keywords:** idiopathic intracranial hypertension, malignant leptomeningeal melanoma, neurology, neurosurgery, pathology‐laboratory medicine

## Abstract

A critical clinical consideration, in addition to other common risk factors predisposing individuals to idiopathic intracranial hypertension (IIH), involves the potential co‐occurrence of increased intracranial pressure and elevated cerebrospinal fluid protein levels in the presence of underlying malignancies. Primary diffuse leptomeningeal melanomatosis, an exceptionally rare condition with few reported cases in the pediatric population, illustrates this scenario. Timely decision‐making based on clinical suspicion to perform a biopsy and involving a skilled pathologist for accurate reporting are essential steps toward achieving a definitive diagnosis.

**Abbreviations**
ACE: angiotensin‐converting enzyme
CNS: central nervous system
CSF: cerebrospinal fluid
CT: computed tomography
DWI: diffusion‐weighted imaging
ICP: intracranial pressure
IHC: immunohistochemical
MRI: magnetic resonance imaging
PDLM: primary diffuse leptomeningeal melanomatosis
WHO: World Health Organization

## 1 Introduction

Primary CNS malignant leptomeningeal melanoma is an extremely rare and aggressive neoplasm especially in children [1] with a global incidence of one case per 20 million individuals [2]. Malignant melanoma is a potentially fatal form of cancer that develops from specialized pigment cells called melanocytes [3]. In humans, central nervous system (CNS) melanocytes are usually most concentrated in the leptomeninges over the ventral aspect of the medulla oblongata [4, 5]. In this location, the pigmentation is often visible macroscopically. As with all melanocytes, these cells are of neural crest origin, developing from normal melanoblasts [5]. Primary CNS malignant melanoma shows aggressive and poor prognosis, and unfortunately, there is no effective treatment for it [6].

Patients typically present with hydrocephalus, cranial nerve palsies, seizures, behavioral abnormalities, and cerebellar dysmetria [7, 8].

## 2 Case History

A 10‐year‐old boy with no previous medical history and no developmental delays presented with headaches and blurred vision for 2 weeks. The patient also reported a history of chickenpox 2 weeks prior to the onset of symptoms. The symptoms gradually worsened, and he developed double vision, for which underwent ophthalmological assessments. He was referred to the pediatric neurology clinic due to papilledema and was admitted for further evaluation. An initial brain CT scan revealed no space‐occupying lesion, so a brain MRI and brain MRV were performed, which did not reveal any structural abnormalities.

## 3 Methods

**FIGURE 1:**
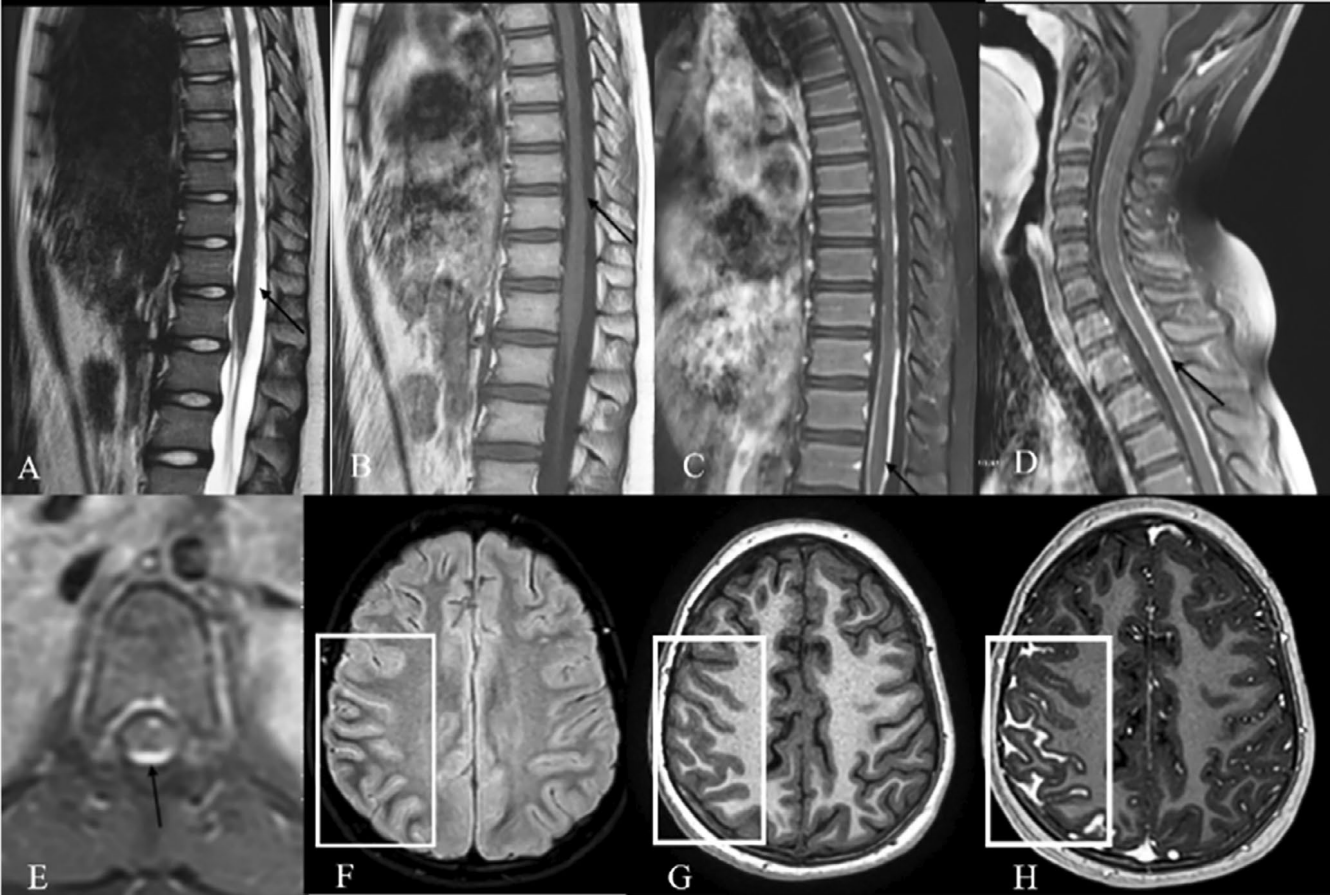
MRI images from the lumbar, thoracic, and cervical spine showing diffuse leptomeningeal. Irregularity and mild thickening (A, sagittal T2‐weighted; B, sagittal non‐contrast T1‐weighted sequences) with significant irregular enhancement on post‐contrast T1‐weighted sequences (C–E, axial views). Brain MRI demonstrated hyperintensity of sulci in the frontoparietal lobes on FLAIR sequence (F), which is obliterated on axial non‐contrast T1 sequence (G) and shows thick enhancement on post‐contrast T1 sequence (H). The involved areas are indicated within the white box.

**FIGURE 2:**
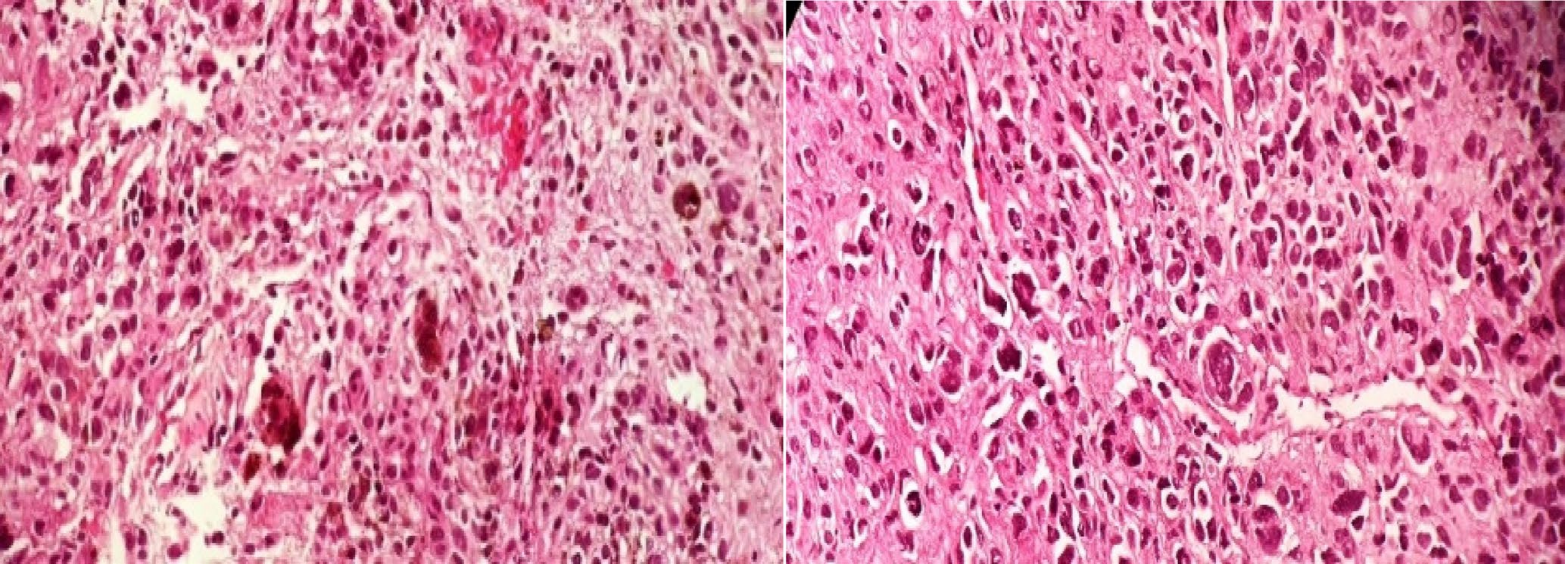
Sections show a hypercellular neoplastic growth composed of pleomorphic epithelioid cells with prominent nucleoli arranged in sheets. Tumor cells also exhibit focal melanin production, hyperchromasia, and formation of atypical giant cells. Scattered mitotic activity is also noted (magnification, ×400, H&E stain).

A lumbar puncture (LP) was performed to assess cerebrospinal fluid (CSF) pressure, which was estimated to be around −45 cm H_2_O, and cerebrospinal fluid cytology was negative for malignant cells. He was prescribed acetazolamide and topiramate and was discharged after 7 days with a diagnosis of idiopathic intracranial hypertension (IIH). The patient was asymptomatic for about 2 weeks but was readmitted with the same symptoms of headache, blurred vision, vomiting and hemiparesis on the left side of his body. A new brain CT without contrast revealed no new findings. Intracranial pressure (ICP) was measured above 50 cm H_2_O, with high CSF opening pressure and high CSF protein levels. Brain and spine MRI with and without gadolinium showed abnormal disseminated leptomeningeal enhancement throughout the neuraxis (Figure 1). A whole‐body scan was normal. No abnormal findings were observed in the spiral chest CT or abdominal and pelvic ultrasound. CSF smear showed many intact and degenerated mononuclear inflammatory cells, including lymphocytes and monocytes. RBCs and a few atypical monocytoid cells were also seen. No obvious malignant cells were observed. Bone marrow aspiration and biopsy were negative for malignancy. The patient developed hydrocephalus during admission, presented with loss of consciousness and underwent ventriculoperitoneal shunting in an emergent setting. Afterward, biopsy was taken from the leptomeninges of the lumbar area in an elective setting. The patient was discharged without neurological defection; the initial histopathology results, mature melanocytes without malignant malformation were reported, and due to disseminated leptomeningeal enhancement, follow‐up was recommended. Four months later, he was admitted to the emergency department with progressive weakness of all four limbs. The weakness started from the upper limb and gradually progressed to the lower limb. He also mentioned nausea and vomiting since 10 days ago. The function of the cerebral shunt was normal. On new brain and spine MRI, very thick leptomeningeal enhancement was shown all through neuroaxis. In particular, compression of the cervical spinal cord was detected. He underwent extensive cervicothoracic osteoplastic laminectomy and duraplasty for cord decompression. During the surgery, a thick black layer was observed covering the spinal cord. Biopsy was taken from the black layer, and the histopathological diagnosis was primary diffuse leptomeningeal melanomatosis (Figure 2).

Due to the insufficient amount of cerebrospinal fluid obtained, a tissue biopsy was performed, which tested negative for the NRAS Q61K mutation.

## 4 Outcome and Follow‐Up

Post‐operatively, the strength of the patient's limbs did not improve. Over the next few days, the patient developed seizure and respiratory distress. He was planned for chemotherapy, but unfortunately he succumbed to death, 8 months after the onset of symptoms.

## 5 Discussion

Leptomeningeal melanomatosis is the name for the rare malignant transformation of leptomeningeal melanocytes [9].

Headache (46%), nausea or vomiting (37%), back or neck discomfort (24%), and weakness (22%) are the most prevalent symptoms. Additional features include spinal cord compression, hydrocephalus, convulsions, ataxia, cranial nerve palsies, cerebral hemorrhage, and neuropsychiatric disorders [10]. Our patient presented with headache, blurred vision, vomiting, hemiparesis, and high CSF pressure and high CSF protein levels, while cerebrospinal fluid cytology was negative for malignant cells. Initially, we suspected IIH (Idiopathic Intracranial Hypertension) because the results of the first tests and imaging did not support the presence of infection or malignancy. However, due to the worsening of symptoms and lack of response to treatment, the patient was reexamined. A new MRI showed very thick leptomeningeal enhancement throughout all neuroaxis, with particular compression of the cervical spinal cord. Following a spinal cord biopsy and pathology report, diffuse leptomeningeal melanomatosis was ultimately identified. Immunohistochemical (IHC) staining showed immunoreactivity of S100, NSE, and SOX10.

Studies have shown that S‐100 is present in human neoplasms originating from Schwann cells and melanocytes and can be detected using immunohistochemistry in both primary and metastatic melanocytic malignancies [11]. Primary diffuse leptomeningeal melanomatosis (PDLM) may mimic other infectious, neoplastic, and autoimmune diseases such as viral encephalitis, meningitis, lymphoma, neurosarcoidosis, and tuberculosis, further complicating the diagnosis [12].

According to Hayward's guide for classification based on clinical findings to diagnose primary CNS melanoma, six factors are important: Absence of malignant melanoma tumor outside the CNS; cranial or spinal involvement with the leptomeninges; spinal lesions that are intramedullary; hydrocephalus; solitary intracerebral lesion; and pituitary or pineal gland tumor [13].

In this 10‐year‐old boy's examination, there was no sign of a mole or lesion indicative of skin and nail melanoma. According to the 2021 World Health Organization Classification of Melanocytic Tumors of the Central Nervous System, the classifications include diffuse meningeal melanocytic neoplasms, meningeal melanocytosis, meningeal melanomatosis, meningeal melanocytoma, and meningeal melanoma [14].

Diagnosing this condition has long been a challenge for clinicians.

We must consider that onset of PDLM can be associated with rising intracranial pressure (ICP) and may be mistaken for idiopathic intracranial hypertension (IIH) [15].

In our case report, IIH was initially considered the primary differential diagnosis based on the negative results of initial investigations. However, due to symptom recurrence and disease progression, a biopsy was repeated, leading to the definitive diagnosis of PDLM.

Among the important differential diagnosis of hydrocephalus is leptomeningeal disease (LMD). LMD represents an advanced complication typically associated with systemic malignancies and results from multifocal metastatic infiltration of leptomeningeal structures, including the pia mater, arachnoid mater, and subarachnoid space. It can manifest clinically as hydrocephalus.

The diagnosis of LMD is definitively confirmed through cerebrospinal fluid cytology, which detects malignant cells [16].

Hydrocephalus is a broad category of conditions caused by impaired circulation or absorption of cerebrospinal fluid(CSF), or, in rare cases, increased CSF production such as in choroid plexus papilloma. It is not a specific disease [17].

**FIGURE 3:**
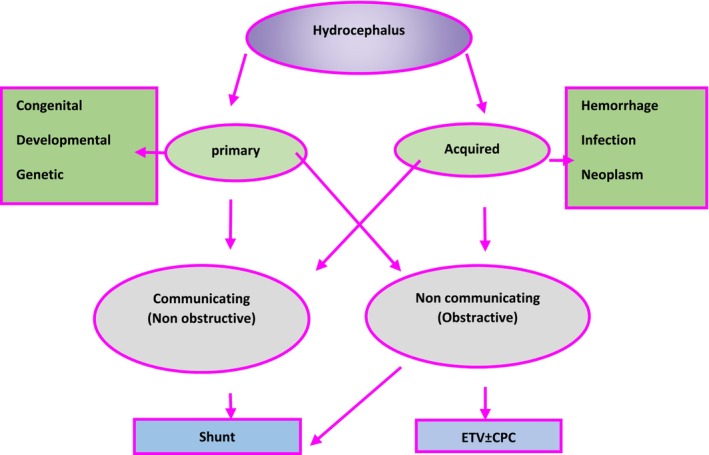
Classification and treatment of hydrocephalus. The figure includes cerebrospinal fluid (CSF) dynamics, endoscopic third ventriculostomy (ETV), and choroid plexus coagulation (CPC).

Treatment options for hydrocephalus can be classified as temporary or permanent. Temporary treatments include procedures such as CSF puncture, external ventricular drainage, ventricular access devices, or ventricular subgaleal shunts [18]. In this case, due to the development of hydrocephalus and loss of consciousness, emergent ventriculoperitoneal shunting was performed (Figure 3).

## 6 Conclusion

When there is elevated intracranial pressure (ICP), performing an MRI with contrast is recommended. If pia arachnoid enhancement is observed, melanomatosis should be considered as one of the possible differential diagnoses. Although rare, melanosis is an important consideration in the differential diagnosis of pia arachnoid enhancement. In summary, primary diffuse leptomeningeal melanomatosis (PDLM) presents a diagnostic and therapeutic challenge due to its rarity in the pediatric population and ambiguous presenting symptoms. The only neurosurgical interventions available are biopsy to obtain a diagnosis and cerebrospinal fluid (CSF) diversion for hydrocephalus. In the event that PDLM is suspected and the initial biopsy yields no results, a timely decision based on clinical and radiological suspicion to perform a biopsy, along with a skilled pathologist for proper reporting, is the best way to reach a definitive diagnosis. Early diagnosis offers the best chance for effective treatment. Meningeal melanomatosis should be considered a potential cause in cases of rising intracranial pressure (ICP). The primary goal of this case report is to raise awareness of this rare disease and emphasize the importance of timely diagnosis. Early detection can help prevent potential complications, such as hydrocephalus and paralysis, Through prompt neurosurgical interventions or shunt placement.

## Author Contributions

**Shiva Sareh:** methodology, project administration, visualization, writing – original draft. **Zohreh Habibi:** conceptualization, data curation, investigation, project administration, supervision. **Mohammad Vasei:** methodology, project administration, validation, writing – original draft. **Moeinadin Safavi:** methodology, project administration, software, writing – original draft. **Alieh Safari Sharari:** methodology, project administration, validation, writing – original draft. **Neda Pak:** supervision, validation, writing – original draft, writing – review and editing. **Golazin Shahbodagh khan:** conceptualization, data curation, supervision, writing – review and editing. **Mahmoudreza Ashrafi:** conceptualization, methodology, project administration, resources, software, supervision. **Morteza Heidari:** conceptualization, data curation, supervision, validation.

## Ethics Statement

All procedures performed in studies involving human participants were in accordance with the ethical standards of the institutional and/or national research committee and with the 1964 Helsinki declaration and its later amendments or comparable ethical standards (IR.TUMS.CMHC.REC.1403.016).

## Consent

Written informed consent was obtained from the patient's parents to publish this report in accordance with the journal's patient consent policy.

## Conflicts of Interest

The authors declare no conflicts of interest.

## Data Availability

Data available on request due to privacy/ethical restrictions.
